# Evaluation of heavy metal-induced responses in *Silene vulgaris* ecotypes

**DOI:** 10.1007/s00709-019-01384-0

**Published:** 2019-05-01

**Authors:** Ewa Muszyńska, Mateusz Labudda, Iwona Kamińska, Mirosława Górecka, Magdalena Bederska-Błaszczyk

**Affiliations:** 1 Department of Botany, Faculty of Agriculture and Biology, Warsaw University of Life Sciences-SGGW, Nowoursynowska 159, Building 37, 02-776 Warsaw, Poland; 2 Department of Biochemistry, Faculty of Agriculture and Biology, Warsaw University of Life Sciences-SGGW, Nowoursynowska 159, Building 37, 02-776 Warsaw, Poland; 3 Unit of Botany and Plant Physiology, Institute of Plant Biology and Biotechnology, Faculty of Biotechnology and Horticulture, University of Agriculture, Al. 29-Listopada 54, 31-425 Krakow, Poland

**Keywords:** Heavy metal toxicity, Photosynthetic apparatus, ROS metabolisms, Non-enzymatic antioxidants, Microscopic imaging, Shoot culture

## Abstract

*Silene vulgaris* is a pseudometallophyte that spontaneously occurs in various ecological niches. Therefore, three ecotypes of this species representing calamine (CAL), serpentine (SER), and non-metallicolous (NM) populations were investigated in this study. Owing to the presence of Pb or Ni ions in natural habitats from metallicolous populations originated, we used these metals as model stressors to determine the survival strategy of tested ecotypes and analyze metal distribution at various levels of organism organization. We focused on growth tolerance, non-enzymatic antioxidants, and photosynthetic apparatus efficiency as well as anatomical and ultrastructural changes occurred in contrasting ecotypes exposed in vitro to excess amounts of Pb^2+^ and Ni^2+^. Although Ni application contributed to shoot culture death, the study revealed that the mechanisms of Pb detoxification differed between ecotypes. The unspecific reaction of both metallicolous specimens relied on the formation of effective mechanical barrier against toxic ion penetration, while the Pb appearance in the protoplasts led to the activation of ecotype-specific intracellular defense mechanisms. Hence, the response of CAL and SER ecotypes was almost unchanged under Pb treatment, whereas the reaction of NM one resulted in growth disturbances and physiological alternations. Moreover, both metallicolous ecotypes exhibited increase generation of reactive oxygen species (ROS) in leaves, even before the harmful ions got into these parts of plants. It may implicate the potential role of ROS in CAL and SER adaptation to heavy metals and, for the first time, indicate on integral function of ROS as signaling molecules in metal-tolerant species.

## Introduction

As a consequence of anthropogenic activities, excess amounts of heavy metals (HMs) have become a global problem. Among them, lead (Pb) and nickel (Ni) are one of the most important contaminants that increase concentration in the environment that are associated with calamine and serpentine ore exploitation and processing. Although Pb might naturally occur in plants, until now, it has not been shown to play any essential roles in their metabolism (Kabata-Pendias 2011). On the contrary, Ni is considered as trace element for some species as a cofactor of various enzymes (Rughani et al. 2016). Despite it, both of them are highly toxic to all living organisms when they occur in elevated concentrations. In vascular plants, Pb and Ni ions can negatively influence morphophysiological processes. Like other HMs, they cause photosynthesis disturbances related to photosynthetic apparatus damages (Rajpoot et al. 2016; Piwowarczyk et al. 2018; Ghori et al. 2019). HMs impact on this basic process may be also shown in decrease of photosynthetic pigment content and/or chlorophyll *a*/*b* ratio due to the inhibition of enzymes involved in chlorophyll biosynthesis as well as disorganization of organelle ultrastructure (Gill et al. 2012; Kumar and Prasad 2018). Therefore, the analysis of photosynthetic pigment content, chlorophyll *a* fluorescence, and chloroplast ultrastructure allows the comprehensive assessment of plant physiological status under stress conditions (Ritchie and Mekjinda 2016; Muszyńska et al. 2017).

Reactive oxygen species (ROS) are constantly produced at low level in non-stressed cells in their chloroplasts and mitochondria as well as by exocellular, membrane-bound, or cytoplasmic enzymes involved in redox reactions (Michalak 2006; Waszczak et al. 2018). In turn, enhanced ROS formation appears in stress conditions when the balance between their generation and scavenging is disturbed (Malar et al. 2014). ROS can oxidize lipids, proteins, or nucleic acids what precede lipid peroxidation, ion leakage, or damage of cell ultrastructure (Maestri et al. 2010; Yadav 2010; Emamverdian et al. 2018). Therefore, HMs application implicates the multiple disorders that resulted not only from direct impact of these toxic ions on various processes but also from additional secondary consequences of oxidative stress, which ultimately lead to the activation of programmed cell death pathways and organism death (Ghori et al. 2019). For this reason, the influence of HMs on plant physiology is also manifested by changes in ROS scavengers preventing deleterious ROS impact on cellular functions (Rajpoot et al. 2016). It follows that stress conditions often induce the synthesis of various antioxidant molecules, among which some non-enzymatic compounds should be mentioned. One of them is phenols that show an antioxidant properties due to the high tendency to bind both HMs and free radicals (Michalak 2006; Kumar and Prasad 2018). These secondary plant metabolites are divided into several groups dependently on their structure. Some of them are highly widespread in plant kingdom, while the others are specific for family or even species (Cheynier 2012; Kisa et al. 2016; Vidot et al. 2019). The protective role of phenols could be explained by their diversified levels depending on stress conditions (Kováčik and Klejdus 2008; Marguez-Garcia et al. 2009; Manquián-Cerda et al. 2018; Muszyńska et al. 2019). Additionally, excess amounts of ROS can be removed by reduced glutathione (GSH). It is a multifunctional cellular thiol, which plays a widespread role in plants during HMs exposure (Wójcik and Tukiendorf 2014; Ghori et al. 2019). GSH not only participate in ROS quenching, but also may form complexes with metal ions in the cytoplasm (Tian et al. 2011). Furthermore, it acts as a precursor for the synthesis of phytochelatins that are cysteine-rich metal-binding peptides (Yadav 2010; Yuan et al. 2015).

Studies on plant response and adaptation to HMs have been intensively conducted by numerous scientific centers (Ovečka and Takáč 2014; Wójcik and Tukiendorf 2014; Rajpoot et al. 2016). Nevertheless, there are only a few research on metal-tolerant representatives of the same species but growing in at least three different, very restrictive habitats that exert severe selection pressures. Through microevolutionary processes, species colonizing areas enriched with HMs are altered ecotypes with specific morphological, anatomical, and physiological traits enabling them to survive in this extremely hostile environment, and thus, they can differ from the individuals living in non-contaminated areas (Muszyńska et al. 2019). Accordingly, post-industrial areas are attractive for researchers because they can observe how nature deals with the harsh conditions and explore adaptive processes of the organisms. *Silene vulgaris* (Caryophyllaceae) deserves particular attention in this context. This species is a specific part of polish plant communities growing on waste deposits created after mining and processing of lead and zinc ores in the Olkusz Ore-bearing Region as well as nickel ores in the Lower Silesia. Furthermore, it occurs commonly in whole Europe in areas unpolluted with HMs. Therefore, three ecotypes of *S. vulgaris* representing calamine, serpentine, and reference non-metallicolous populations were selected for the purpose of our experiment. Owing to the presence of Pb^2+^ and Ni^2+^ in natural habitats from metallicolous populations originated, we used these metals as model stressors. In the current study, we compared growth tolerance and physiological status as well as anatomical and ultrastructural alterations that occurred in contrasting ecotypes exposed in vitro to excess amounts of Pb or Ni ions. We applied the microscopic imaging techniques to determine specific and conservative defense mechanisms against toxic metals and analyze their distribution at various levels of organism organization. Taking into account that little is known about the relationship between HMs and ROS metabolism in metallophytes, we hypothesized that oxidative stress, resulted from HMs treatment, might alter *Silene* response to toxic ions in various ways. We assumed that tolerance in metallicolous ecotypes relies on the maintenance of steady-state ROS level by the production of genotype-specific secondary metabolites that participate also in HMs binding.

## Materials and methods

### Culture condition and heavy metal treatment

Three ecotypes of *Silene vulgaris* (Moench) Garcke from Caryophyllaceae family representing metallicolous polish populations from (1) calamine waste heap in Bolesław, Olkusz Ore-bearing Region, the Śląsko-Krakowska Upland (described further as CAL), (2) serpentine waste heap in Wirki, the Gogołów-Jordanów serpentine massif, Lower Silesia (described further as SER), and (3) non-metallicolous population that originated from the University of Warsaw Botanic Garden (described further as NM) were used in this study. The cultures of each ecotype were previously multiplicated according to the protocol proposed by Muszyńska et al. (2019) on basal MS medium (Murashige and Skoog 1962) containing 20.0 g L^−1^ sucrose, 0.5 mg L^−1^ NAA (1-naphtaleneacetic acid), and 5.0 mg L^−1^ BA (6-benzylaminopurine). The pH of medium was adjusted to 5.8 prior to solidification with agar (0.9% *w*/*v*). Test cultures to investigate the plant response to HMs treatment were established by placing 20-mm-long apical fragments of shoots on above-described proliferation medium supplemented with 33 μM lead nitrate or 1.0 mM nickel(II) sulfate. The applied concentrations of HMs were adequate to the content of soluble fractions of Pb^2+^ and Ni^2+^ directly absorbed by plants in areas from which seed samples to start in vitro culture were previously taken (Koszelnik-Leszek 2007; Muszyńska et al. 2017). As a control, medium without HM salts was used. HMs were added to medium, prior to autoclaving, and pH was adjusted to 5.8. Five microcuttings per 200-mL flask were explanted on the respective media and each flask contained 50 mL of culture medium. Six replicates (flasks) per each treatment were used, which correspond to at least 30 explants. Cultures were maintained in a growth chamber MLR-350 (Sanyo, Tokyo, Japan) at 24 °C, under 16-h photoperiod (irradiance 80 μmol m^−2^ s^−1^). The experiment lasted 4 weeks and was repeated four times.

### Morphological parameter assessment

After 4 weeks of culture, the microplantlet growth and vitality was assessed. The obtained shoots were counted and multiplication coefficient (MC) was calculated as the total number of regenerated shoots per primary explant. The percentage of rooted shoots and the number of spontaneously regenerated roots were evaluated. Plant material was dried in 105 °C to the constant mass and weighted afterwards for determination of dry matter. Growth tolerance index (in %) was ascertained for entire microplantlets, i.e., rooted shoots, using the formula:

GTI = (mean dry weight of shoots and roots developed on medium supplemented with metal salt / mean dry weight of shoots and roots developed on medium without metal salt) × 100%.

### Heavy metal detection

#### Cryo-SEM-EDX

Evaluation of native-state samples was performed using cryo-scanning electron microscopy. Chosen leafs were cut using sterile surgical blade and cross-sectioned at about one-third of their length. Samples were immediately mounted on cryo-holder using OCT compound mixed with colloidal graphite. Mounted material was immersed in liquid nitrogen using Quorum device and quickly transferred into the cryo-preparation chamber at − 140 °C. Samples were sputtered with platinum and introduced to scanning electron microscope chamber (Auriga 60, Zeiss). The morphology of cross-sectioned surfaces were observed at − 140 °C and 2 kV of acceleration voltage using SE2 and InLens detectors. Elemental composition and distribution analyses were performed at 20 kV of acceleration voltage using Oxford detector. Metal concentrations were expressed in percentage by weight (wt%).

#### Tissue localization

The presence of HMs in tissue of *Silene* leaves was demonstrated using the fluorescent indicator Phen Green SK diacetate salt (C_37_H_21_Cl_2_N_3_O_8_, *N*-(6-Methoxy-8-Quinolyl)-p-Toluenesulfonamide, Life Technologies, USA). It is cell permeable and sensitive to a range of transition elements including Pb^2+^. The series of handmade leaf sections were stained in 50 μM fluorescent probe for 1 h at room temperature. As a control, unstained sections were used. After incubation, sections were washed three times by PBS buffer and observed under Leica TCS SP5II laser scanning microscope (Leica Microsystems CMS, Wetzlar, Germany). The excitation/emission range of 488/505–530 nm was chosen to visualize the presence of metals.

#### Scanning transmission electron microscopy analysis

Ultrastructural analyses were performed on high-pressure frozen tissue samples. Chosen leafs were cut using sterile surgical blade and the 5 mm of diameter cuts was collected using skin biopsy punch at central region of leafs. Each sample was put into the type A planchettes with yeast paste used as a cryoprotecting agent. Samples were vitrified at 2100 bar using Leica HPM 100. Frozen samples were subsequently transferred under liquid nitrogen to cryo-vials containing frozen freeze-substitution medium (95% acetone/1% osmium tetroxide/0.1% uranyl acetate) and placed in Leica AFS device for automatic freeze substitution process. The process parameters were set as follows: − 140 to − 80 °C for 120 min, − 80 to − 20 °C for 60 min, − 20 to + 4 °C for another hour (modified method published previously; Bobik et al. 2014). After reaching 4 °C, the samples were transferred to pure acetone for 5 min, infiltrated with agar resin, and polymerized at 60 °C overnight. Ultrathin (~ 70 nm thick) sections were prepared using Leica UC7 ultramicrotome. Sections were collected on 200 mesh copper TEM grids. Samples were observed using Auriga 60 scanning electron microscope by means of scanning transmission electron microscopy (STEM) detector at 20 or 30 kV of acceleration voltage. The elemental composition was determined using Oxford detector.

### Oxidative stress determination

#### Total ROS detection in tissues with confocal laser scanning microscope (CLSM)

Overall accumulation of ROS was monitored microscopically using widely applied reagents—CellROX® Oxidative Stress Reagents (Life Technologies, USA). These cell-permeate dyes are non-fluorescent while in a reduced state and exhibit strong fluorogenic signal upon oxidation by ROS. The signals from CellROX® Deep Red and CellROX® Orange Reagents are localized in the cytoplasm, whereas from CellROX® Green Reagent in the nucleus and mitochondria. Stock solution of proper dye in DMSO was diluted by 0.1 M PBS buffer (pH = 6.9) to final concentration of 5 mM. The series of handmade leaf sections were stained in each dye for 30 min at 37 °C. As a control, unstained sections were used. After incubation, sections were washed three times by PBS buffer and observed under Leica TCS SP5II laser scanning microscope (Leica Microsystems CMS, Wetzlar, Germany). The excitation/emission range of 644/665–677 nm, 545/565–600 nm, and 485/510–530 nm were chosen for Red, Orange, and Green Reagents, respectively.

#### Ultrastructural H_2_O_2_ localization

Accumulation of H_2_O_2_ in leaves was visualized by transmission electron microscopy using histochemical method based on generation of cerium perhydroxide precipitates (Bestwick et al. 1997). Ten fragments of the middle part of leaf blade, approximately 3 × 3 mm in size from each treatment, were incubated in freshly prepared 5 mM CeCl_3_ in 50 mM MOPS [3-(N-morpholino)propanesulfonic acid] at pH 7.2 for 1 h. The control for H_2_O_2_ localization was run without the addition of CeCl_3_. Then, samples were fixed according to Karnovsky (1965) for 3 h, rinsed four times in 0.1 M cacodylate buffer, and post-fixed in 2% (*v*/*v*) OsO_4_ for 2 h at 4 °C. After dehydration in an ethanol series and substitution by propylene oxide, samples were embedded in EPON epoxy resin and polymerized at 60 °C for 24 h. Ultra-thin sections (90 nm thick) were prepared with UCT ultramicrotome (Leica Microsystems), contrasted with uranyl acetate (5 min) followed by incubation in lead citrate (0.5 min), and examined in an FEI 268D “Morgagni” transmission electron microscope.

#### H_2_O_2_ content

The H_2_O_2_ content was measured according to Junglee et al. (2014). One hundred milligrams of fresh shoots was homogenized in 1.75 mL of medium containing 1 mL of 0.1% (*w*/*v*) trichloroacetic acid (TCA), 0.5 mL of 1 M KI, and 0.25 mL of 10 mM K/Na-phosphate buffer (pH 5.8). The homogenates were centrifuged (4 °C, 15 min, 15,000×*g*) and the supernatants were incubated in the dark for 20 min at room temperature. After incubation, the samples were centrifuged once again (5 min, 16,000×*g*), and the absorbance was measured at 350 nm. The H_2_O_2_ content was estimated from a standard curve and calculated per gram of fresh shoot weight (FW).

#### Lipid peroxidation

The 2-thiobarbituric acid reactive substance (TBARS) method according to Heath and Packer (1968) was used. Plant samples were homogenized in 1 mL of ice-cold 10% (*w*/*v*) TCA containing 0.25% (*w*/*v*) thiobarbituric acid. Homogenates were centrifuged (4 °C, 20 min, 10,000×*g*), and supernatants were collected and incubated for 30 min at 96 °C. Subsequently, samples were cooled in the ice bath and centrifuged (10 min, 10,000×*g*). The absorbance of samples was measured at 532 and 600 nm. The TBARS content was estimated using an extinction coefficient of 155 mM^−1^ cm^−1^ and expressed as μmol TBARS per gram of FW.

### Evaluation of antioxidant properties

#### Reduced glutathione localization

In situ labeling of glutathione (GSH) was carried out with monochlorobimane (MCB) as fluorescent marker. Stock solutions of 100 mM MCB (Molecular Probes, USA) were prepared in dimethyl sulfoxide (DMSO) and stored at − 20 °C. Aliquots were diluted in deionized water to a final concentration of 100 μM. In order to deplete ATP levels and thereby inhibit vacuolar sequestration of glutathione *S*-bimane conjugate (GSB), sodium azide (Sigma-Aldrich, Taufkirchen, Germany) was added to the dye solution at a final concentration of 5 mM. The series of handmade leaf sections were incubated for 15 min in the vacuum and then for 75 min at room temperature. As a control, unstained sections were used. After incubation, sections were washed three times by DMSO buffer and transferred to a drop of deionized water on a microscope slide. GSB fluorescence was imaged using Leica TCS SP5II laser scanning microscope (Leica Microsystems CMS, Wetzlar, Germany). The excitation/emission range of 405/522–555 nm was applied (Hartmann et al. 2003).

#### Secondary metabolite visualization

Handmade cross-sections of fresh leaves were cut with a razor blade and placed in water. The observations were performed under a bright field or UV irradiation. The fluorescence microscope equipped with a U-MNU narrow-band filter cube (Olympus-Provis, Japan) was used for analysis of secondary metabolite autofluorescence.

#### Ultrastructural phenol localization

Accumulation of phenols in leaves was visualized by transmission electron microscopy using the caffeine which caused the condensation of phenolics inside the vacuoles (Mueller and Greenwood 1978). One percent caffeine (*w*/*v*) was added to the Karnovsky (1965) fixative medium and washing 0.1 M cacodylate buffer during fixation. The post-fixation, dehydration, and embedding steps were conducted similarly to above described for H_2_O_2_ localization. The control was run without the addition of caffeine.

#### Phenolic compound concentration

One hundred milligrams of freeze-dried shoot tissue was homogenized with 5 mL of 80% (*v*/*v*) methanol and centrifuged (15 min, 2500×*g*). The concentration of total phenols, phenylpropanoids, flavonols, and anthocyanins was determined according to Fukumoto and Mazza (2000). The supernatant was mixed with 0.1% HCl (in 96% ethanol) and 2% HCl (in water), and 15 min after mixing, the absorbance was recorded. The assay detects the chemical compounds with double bonds in their structure that exhibit the absorbance at 280, 320, 360, and 520 nm, which correspond to total phenols, phenylpropanoids, flavonols, and anthocyanins’ presence, respectively. The chlorogenic acid (total phenols), caffeic acid (phenylpropanoids), quercetin (flavonoids), and cyanidin (anthocyanins) were used as standards for determination of particular phenol group. To specify typically arranged aromatic phenols containing a phenyl group bonded to a hydroxy group (polyphenols), the Folin-Ciocalteu assay was used. Shoot extracts were prepared as described above. Briefly, 20 μL of each methanolic extract was mixed with 1.58 mL of deionized water and 100 μL of Folin-Ciocalteu reagent (POCH, Gliwice, Poland). Samples were incubated at room temperature for 4 min, and next 300 μL of 1 M saturated Na_2_CO_3_ solution was added and the mixtures were incubated at 40 °C for 30 min. The absorbance was read at 740 nm and polyphenol content was quantified as gallic acid equivalent. The gallic acid concentration was counted from a standard curve (1–20 μg). Regardless of the methods, the results were expressed in milligrams of the respective standard equivalents per 100 g of FW.

### Evaluation of photosynthetic apparatus efficiency

#### Photosynthetic pigment concentration

One hundred milligrams of freeze-dried shoot tissue was extracted with 80% acetone with addition of CaCO_3_ in ice-cold conditions. The samples were centrifuged for 15 min at 2500×*g* at 4 °C. The absorbance of chlorophyll *a* (chl *a*), chlorophyll *b* (chl *b*), and carotenoids (car) was recorded at 470, 646, and 663 nm, respectively. The pigment content was calculated according to Wellburn (1994) equations. The content of total chlorophylls (chl *a* + *b*), the ratio of chlorophyll *a* to *b* (chl *a*/*b*), and the ratio of total chlorophylls to carotenoids (chl *a* + *b*/car) were also calculated.

#### Chlorophyll *a* fluorescence

Fluorescence of the chlorophyll *a* was measured with the HandyPea fluorimeter (Hansatech Instruments, UK) after dark adaptation for 30 min (*N* = 5–10). Several parameters were calculated on the basis of the fluorescence curves: *F*_v_/*F*_m_—maximum quantum efficiency of PSII; RC/ABS—density of reaction centers per chlorophyll; *F*_v_/*F*_o_—activity of the water-splitting complex; (1 − *V*_j_)/*V*_j_—measure of forward electron transport; PI—performance index. Area indicates the size of the plastoquinone pool.

### Statistical analyses

Results were subjected to two-way analysis of variance (factors: ecotype and metal treatment) and a post hoc Fisher’s test was used to determine significant differences between means at *P* < 0.05. Statistica 13.3 (StatSoft Inc., Tulsa, OK, USA) was used to carry out the statistical analyses. Results for growth parameters were obtained from 18 replicates. Results for biochemical analysis were obtained from four replicates. Results for microscopic imaging were obtained from ten middle parts of leaves of the third node. Moreover, the microscopic observations of ROS were performed two times—24 h and 4 weeks after heavy metal treatment, while secondary metabolite visualization and physiological status of culture were evaluated at the end of experiment.

## Results

### Culture development in the presence of toxic ions

The influence of Ni ions on *S. vulgaris* cultures was noticed just after 1 week of cultivation when rolling of leaf blades occurred independently of ecotype. Moreover, the yellowing of NM shoots was observed, while the intensive anthocyanin coloration appeared in CAL and SER shoots. Finally, irrespective of tested ecotype, the whole culture died; however, in SER culture, the phytotoxic effects of Ni^2+^ were shown several days later than in other ecotypes. This experimental step was repeated four times and the similar results were obtained, even if previously rooted shoots were put into the Ni-containing medium (data not shown). Therefore, the biometric measurements as well as structural and biochemical analysis had been omitted.

The culture of tested ecotypes proliferated vigorously on medium enriched with Pb ions (Table 1). Nevertheless, multiplication rate of NM shoots on medium containing Pb salt was significantly reduced by approximately 40% in comparison with untreated ones, while multiplication of CAL and SER shoots was not inhibited in this stress condition. The length of NM and SER shoots was affected by the addition of Pb ions to propagation medium and amounted about 12–13 mm. On the contrary, CAL shoots reached similar length of 17–20 mm in both Pb-treated and untreated culture. Moreover, on Pb-containing medium, a significant increase of shoot dry weight occurred in metallicolous ecotypes (i.e., CAL and SER), while no effect of Pb on dry weight content of NM shoots was observed. During cultivation, shoots spontaneously formed adventitious roots; however, the percentage of rooted explants was reduced on Pb-supplemented medium regardless of ecotypes. Therefore, the differences in average number of roots produced by a single shoot were also shown. The application of Pb(NO_3_)_2_ did not influence root dry weight content in NM roots which range from 8.0 to 8.8%. A statistically significant decrease in this parameter under Pb stress was recorded in CAL roots, whereas in SER roots, its increase was noted. Calculation of growth tolerance index (GTI) revealed that the growth of non-metallicolous microplantlets treated with Pb ions was reduced about 25% in comparison with control treatment. On the contrary, the growth of CAL cultures was promoted by the presence of Pb^2+^ or hardly even undisturbed in case of SER ones. For these metallicolous microplantlets, GTI amounted 142% and 94%, respectively (Table 1).

**Table 1:** Growth parameters of *Silene vulgaris* ecotypes (NM—non-metallicolous, CAL—calamine, SER—serpentine) on media containing Pb(NO_3_)_2_

Ecotype	Treatment	MC	Shoot length [mm]	Shoot dry weight content [% FW]	Rooted explants [%]	Root number/explant	Root dry weight content [% FW]	GTI [%]
NM	Control	2.6 ± 1.0 b*	29.1 ± 7.5 a	11.8 ± 2.1 bc	90	5.8 ± 3.2 a	8.8 ± 2.2 ac	n/a
	33 μM Pb	1.6 ± 0.4 c	19.7 ± 4.0 b	12.2 ± 2.0 abc	40	1.7 ± 1.1 b	8.0 ± 0.3 abc	74
CAL	Control	5.2 ± 1.3 a	20.9 ± 4.8 b	10.2 ± 0.6 c	55	2.4 ± 1.3 ab	9.4 ± 0.3 a	n/a
	33 μM Pb	5.0 ± 1.2 a	17.7 ± 4.1 b	14.2 ± 1.2 a	15	0.2 ± 0.1 c	6.8 ± 1.1 c	142
SER	Control	2.2 ± 1.4 bc	33.8 ± 7.1 a	10.6 ± 0.9 c	65	1.6 ± 0.5 b	7.7 ± 0.7 bc	n/a
	33 μM Pb	2.0 ± 0.1 bc	19.8 ± 2.6 b	12.9 ± 1.1 ab	30	0.8 ± 0.5 c	9.6 ± 0.3 a	94

*MC* micropropagation coefficient, *GTI* growth tolerance index, *n/a* not analyzed

*Data present means ± SD. Different letters indicate means that are significantly different at *P* < 0.05 according to two-way ANOVA and post hoc test

### Heavy metal localization

**Fig. 1:**
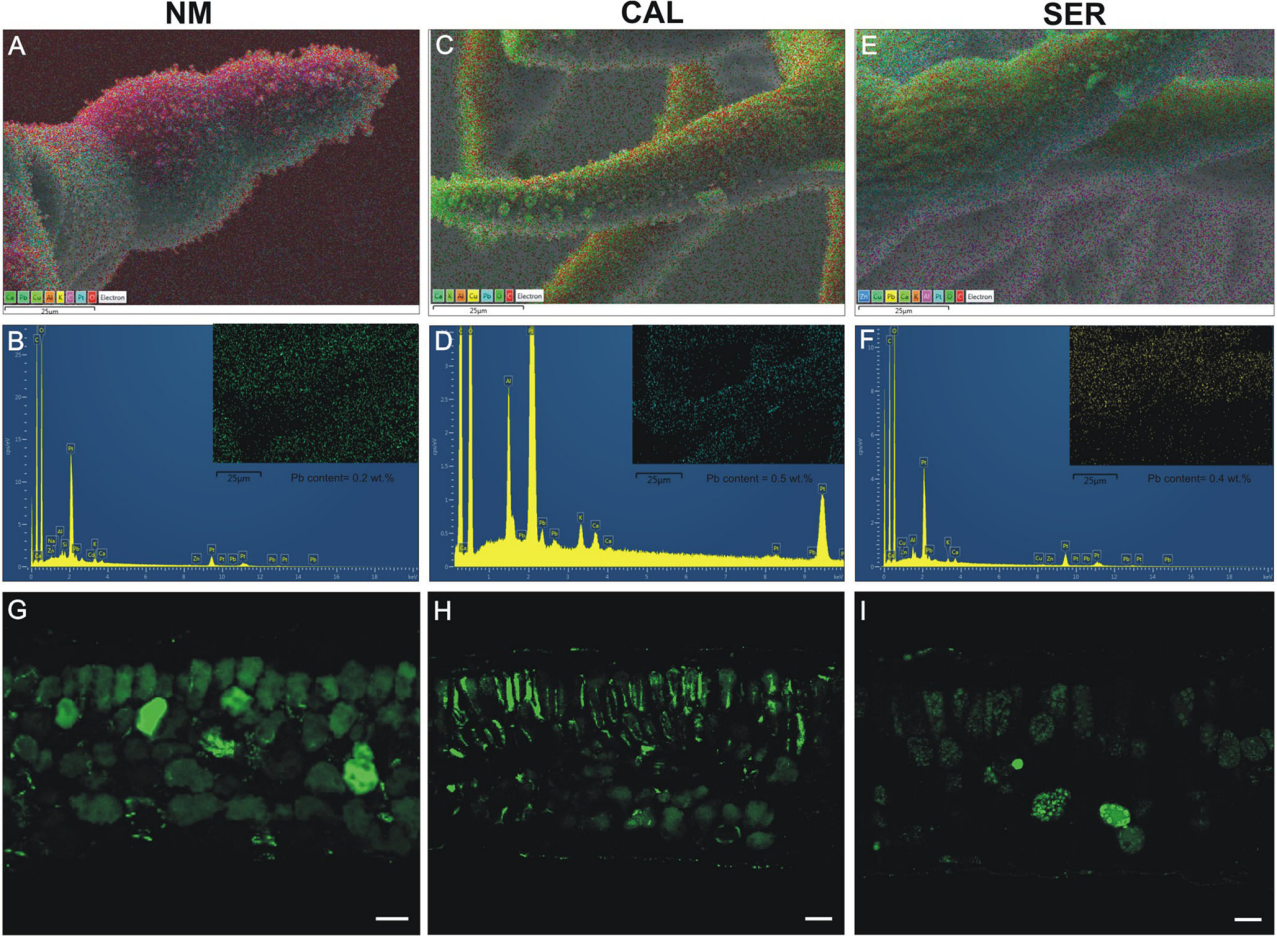
Heavy metal localization in leaves of non-metallicolous (NM), calamine (CAL), and serpentine (SER) ecotypes cultivated on medium enriched with Pb ions. **a**–**f** SEM electron images and corresponding EDX spectra. The colored spots (**b**, **d**, **f**) forming a trichome shape indicated Pb presence in this structure. **g**–**i** Transverse leaf sections stained with fluorescent indicator of metals (Phen Green SK diacetate salt). Green coloration showed metal distribution in leaf tissue. Bar = 25 μm

Independently on analytical method, the presence of metals in tissues was detected only after 28 days of cultivation on medium enriched with Pb^2+^. The cryo-SEM-EDX analysis revealed similar content of Pb^2+^ in leaf surface (0.4 wt%) and vascular bundles (0.3 wt%) of all tested specimens treated with these ions. The most pronounced differences between ecotypes were observed for trichomes (Fig. 1a–f). The lowest Pb concentration was localized in NM trichomes (0.2 wt%; Fig. 1a, b), while the highest in CAL one (0.5 wt%, Fig. 1c, d). The Pb content in SER trichomes reached the intermediate value of 0.4 wt% (Fig. 1e, f). Based on fluorescent staining, the diversified ability of individual ecotypes to accumulate toxic ions in mesophyll was also demonstrated (Fig. 1g–i). In NM leaves, metals were detected in vacuoles of mesophyll cells of the whole leaf blade cross-section (Fig. 1g). For CAL ecotype, ions were noted in the cytoplasm of palisade mesophyll and in the vacuole of spongy one (Fig. 1h), while for SER mainly in vacuoles of palisade mesophyll (Fig. 1i). Furthermore, in CAL leaves, the intensity of fluorescence increased towards the edge of leaf blade, where trichomes are located. Considering ultrastructural metal localization by STEM, the analysis did not provide the expected results and metals were not found in cell compartments.

### ROS detection in *Silene* shoots

#### CLSM investigation

**Fig. 2:**
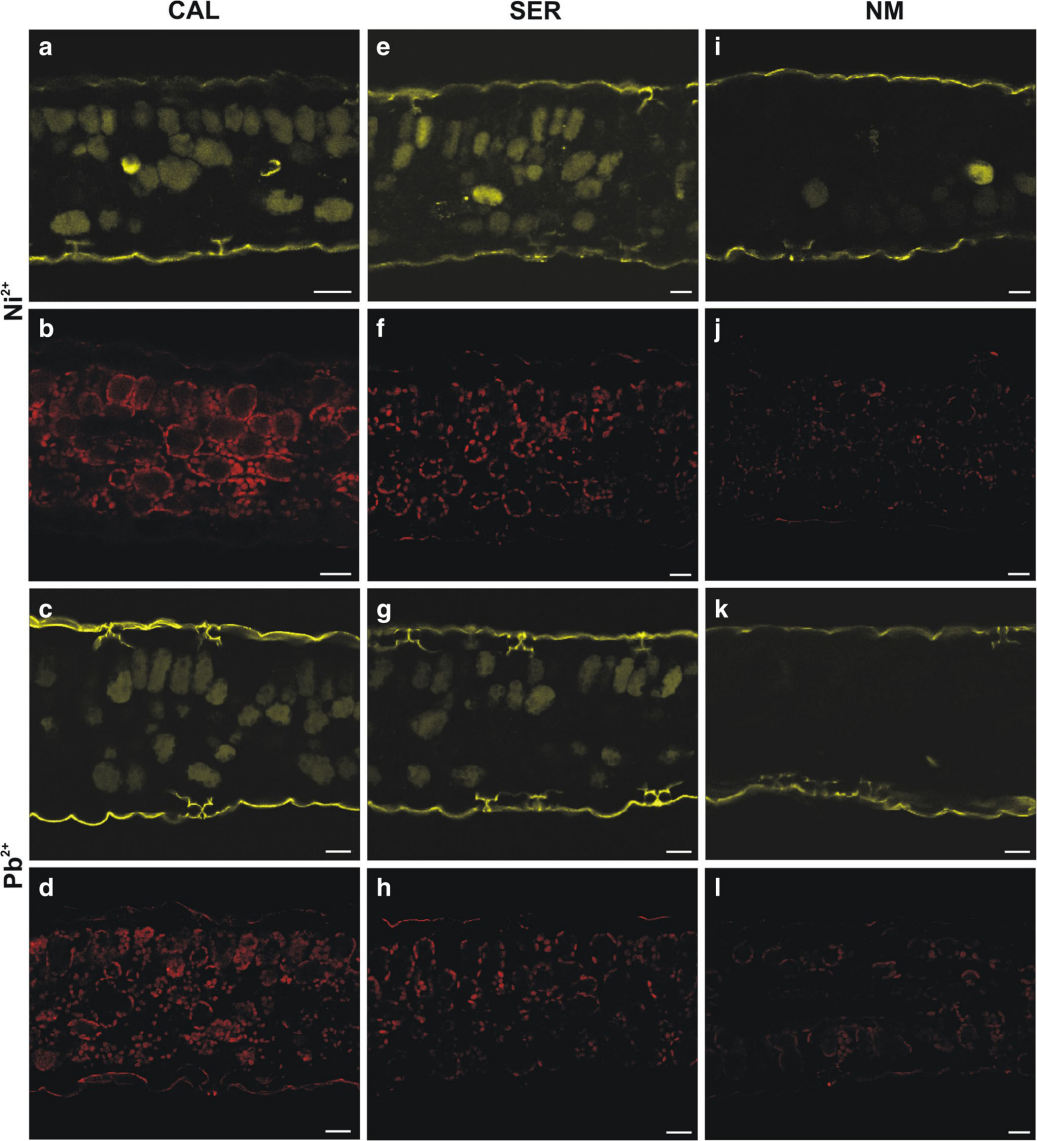
Reactive oxygen species distribution in calamine (CAL; **a**–**d**), serpentine (SER; **e**–**h**), and non-metallicolous (NM; **i**–**l**) *Silene vulgaris* specimens treated with Ni or Pb ions by 24 h. Orange and red coloration indicated the presence of reactive oxygen species in leaf tissue. Fluorescent staining with CellROX® Oxidative Stress Reagents. Bar = 25 μm

**Fig. 3:**
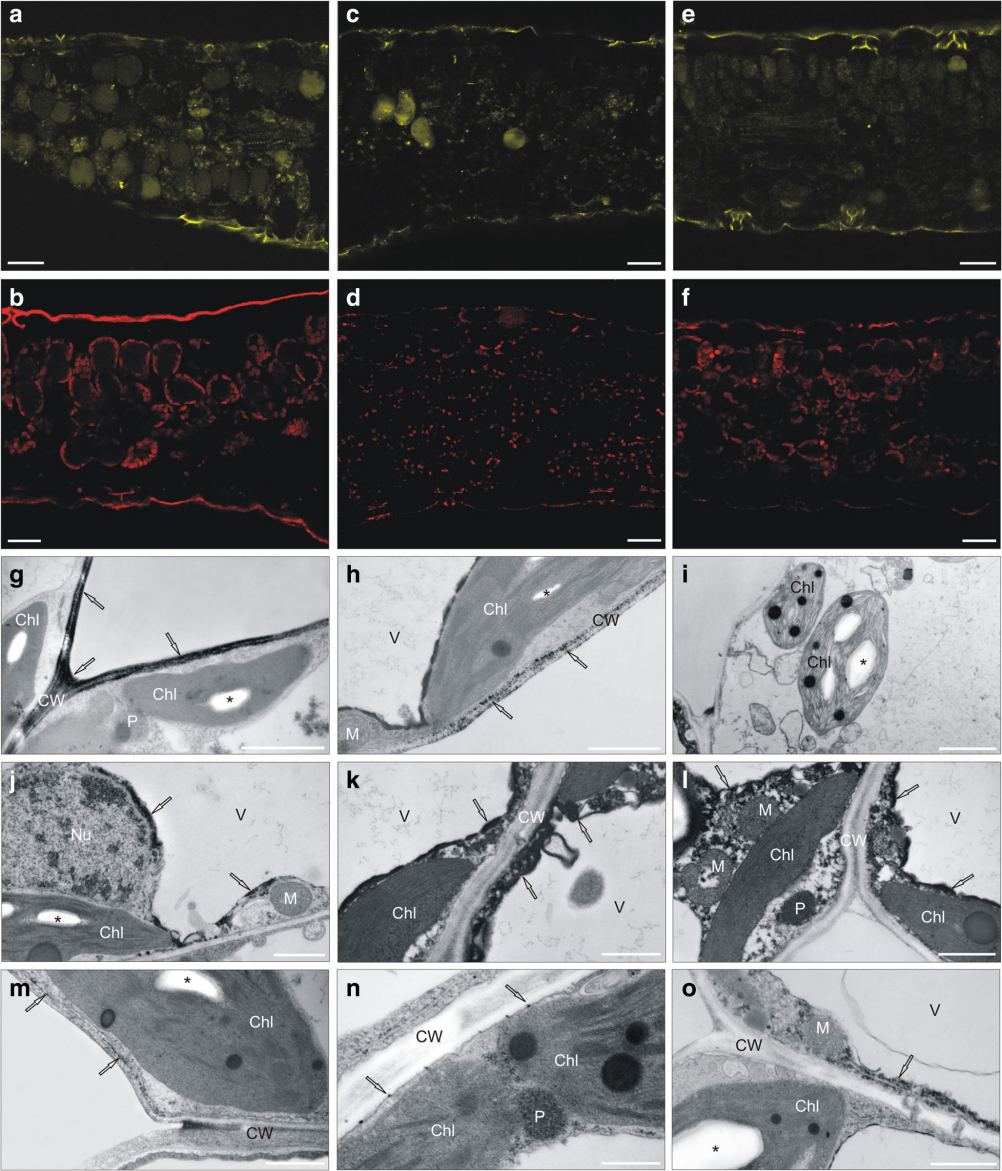
Visualization of reactive oxygen species (ROS) in *Silene vulgaris* leaves growing on medium enriched with heavy metals. **a**–**f** ROS distribution in non-metallicolous (**a**, **b**), calamine (**c**, **d**), and serpentine (**e**, **f**) specimens cultivated in the presence of Pb ions for 28 days. Bar = 25 μm. **g**–**i** Hydrogen peroxide localization in leaf mesophyll cells after 24-h treatment with heavy metals. Abundant cerium perhydroxide dark precipitates, indicating the presence of hydrogen peroxide, were observed in cell walls of calamine specimens treated with Pb ions (**g**) and in serpentine one (**h**) treated with Ni ions. No precipitates were detected in Ni-treated non-metallicolous specimens (**i**), in which many ultrastructural disorders appeared. **j**–**o** Detection of hydrogen peroxide in the cytoplasm of non-metallicolous cells (**j**–**l**), in the cell walls of calamine cells (**m**), and in the plasma membranes (**n**) and the cytoplasm of serpentine cells treated with Pb ions for 28 days. Bar = 2 μm. Chl chloroplast, CW cell wall, M mitochondrion, Nu nucleus, P peroxisome, V vacuole, asterisk starch grain, arrow cerium perhydroxide precipitates

Independently of ecotypes and culture duration, in control specimens, CellROX Reagents staining revealed similar trends of ROS localization in the cross leaf blade section. The ROS signals were concerned to the chloroplasts (Deep Red reagent) or sporadically to the cytoplasm (Orange Reagent). The intensification of fluorescence, indicating excess ROS production, was noted in leaves under heavy metal stress. Neither roots of control specimens nor treated with toxic ions showed intensive changes. After 24 h, the strongest fluorescence was observed in the cytoplasm of CAL parenchyma cells irrespective of applied ions (Fig. 2a–d). In SER leaves, intensive fluorescence of parenchyma was found after Ni^2+^ application, while in Pb-treated leaves, signals were noted mostly in the cytoplasm and chloroplasts of palisade cells (Fig. 2e–h). At the time, NM leaves did not show such intensive ROS accumulation which level in the cytoplasm was comparable in both Ni- and Pb-treated leaves (Fig. 2i–l). After 4 weeks of cultivation in the presence of Pb ions, the general ROS level exhibited the opposite tendency (Fig. 3a–f). The most intensive fluorescent signals, confirming stronger appearance of oxidative stress, were detected in NM leaves (Fig. 3a, b). In this case, the orange signal was located chiefly in the palisade parenchyma cells, while the red fluorescence was observed in the cytoplasm and chloroplasts of both parenchyma cells. On the contrary, in CAL leaves, the weak fluorescent signals were visualized sporadically (Fig. 3c, d). In SER leaves, ROS were localized mostly in the cytoplasm (Fig. 3e, f). Unlike other ecotypes, in Pb-treated SER leaves, the strongly fluorescent red spots occurred. Nevertheless, its initially intensive signal was lost very quickly. Considering CellROX Green Reagent, fluorescent signals were noted only at the end of experiment in mitochondria of SER Pb-treated leaves. This observation corresponded to the electron microscopy study that revealed numerous changes in size and shape of mitochondria from leaves of these specimens. In other tested ecotypes, green signals were reported rarely.

#### Ultrastructural H_2_O_2_ visualization and its concentrations

After 24 h, TEM visualization of H_2_O_2_ showed their abundant presence in the cell walls of CAL and SER ecotypes from Pb- and Ni-enriched medium, respectively (Fig. 3g, h). Taking into account control reaction without the addition of CeCl_3_, it should be assumed that dark deposits observed on the tonoplast of SER cells probably indicated the accumulation of phenolic-like substances. In HM-treated NM ecotype as well as all control specimens, H_2_O_2_ was not detected. Interestingly, many malformation of nucleus and chloroplast ultrastructure such as swollen thylakoids or large plastoglobuli as well as disruption of membranes and increased vacuolization were noticed in Ni-treated NM leaves (Fig. 3i). Such disturbances were not observed in metallicolous leaves under HMs stress. After 28 days, in control NM leaves, H_2_O_2_ was shown only in cells showing apoptosis-like symptoms. Similar reactions occurred in untreated CAL and SER leaves; however, in this case, the number of degraded cells was relatively low. In leaves collected from NM specimens treated with Pb^2+^, abundant cerium perhydroxide precipitates formed in the cytoplasm around nucleus and chloroplasts (Fig. 3j–l). In leaves of metallicolous ecotypes cultivated on Pb-enriched medium, H_2_O_2_ was visualized only in cell walls of both parenchyma cells in case of CAL leaves (Fig. 3m), while in SER leaves, dark precipitates were found in plasma membranes (Fig. 3n) and sporadically in cytoplasm (Fig. 3o).

**Fig. 4:**
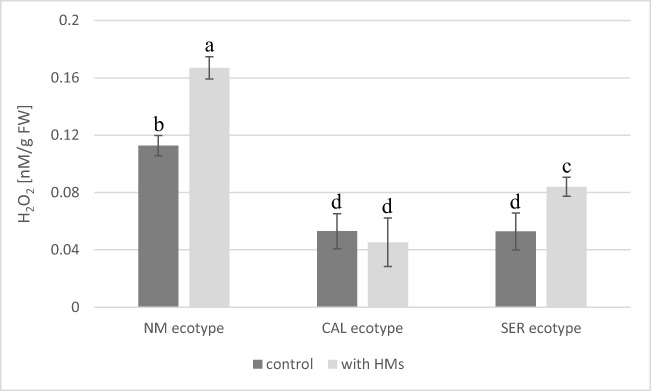
The level of H_2_O_2_ (± SD) in shoots of *Silene vulgaris* ecotypes (NM non-metallicolous, CAL calamine, SER serpentine) cultivated on control medium and enriched with Pb ions

At the end of experiment, spectrophotometric measurement of H_2_O_2_ was performed. Statistical analysis revealed that the level of this molecule depended on both ecotypes and their interaction with HM treatment (Fig. 4). In control NM shoots, the level of H_2_O_2_ was significantly enhanced than in control CAL and SER shoots which presented similar content of this molecule. As a result of Pb^2+^ application, the level of H_2_O_2_ changed only in NM and SER shoots and was about 1.6-fold higher in comparison to their controls. No significant difference in H_2_O_2_ content was observed between control and Pb-treated CAL shoots.

### Effect of Pb ions on lipid peroxidation

**Fig. 5:**
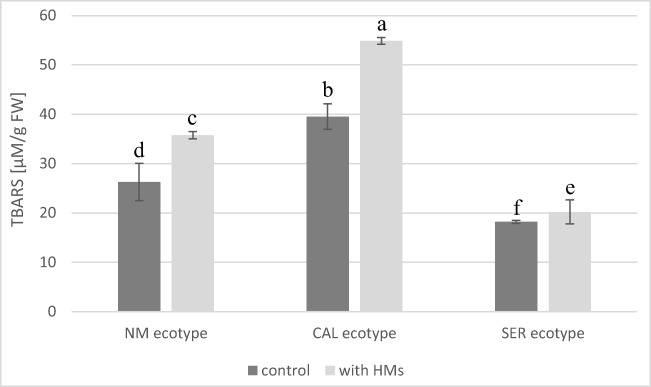
The lipid peroxidation in shoots of *Silene vulgaris* ecotypes (NM non-metallicolous, CAL calamine, SER serpentine) expressed in thiobarbituric acid reactive substance (TBARS) content (± SD)

The lipid peroxidation was estimated by measurement of thiobarbituric acid reactive substance (TBARS) content. It was found that its content depended on all tested factors, i.e., treatment, ecotypes, and their combination. Compering ecotypes, the highest TBARS concentration was ascertained in CAL culture (Fig. 5). Under Pb stress, TBARS level enhanced similarly in NM and CAL shoots about 1.4-fold than in respective controls. In case of SER culture, an increase about 1.1-fold in peroxidation was noted for shoots growing on Pb-supplemented medium as compared to untreated ones.

### Non-enzymatic antioxidants in Pb-treated microcuttings

#### Leaf localization of glutathione

**Fig. 6:**
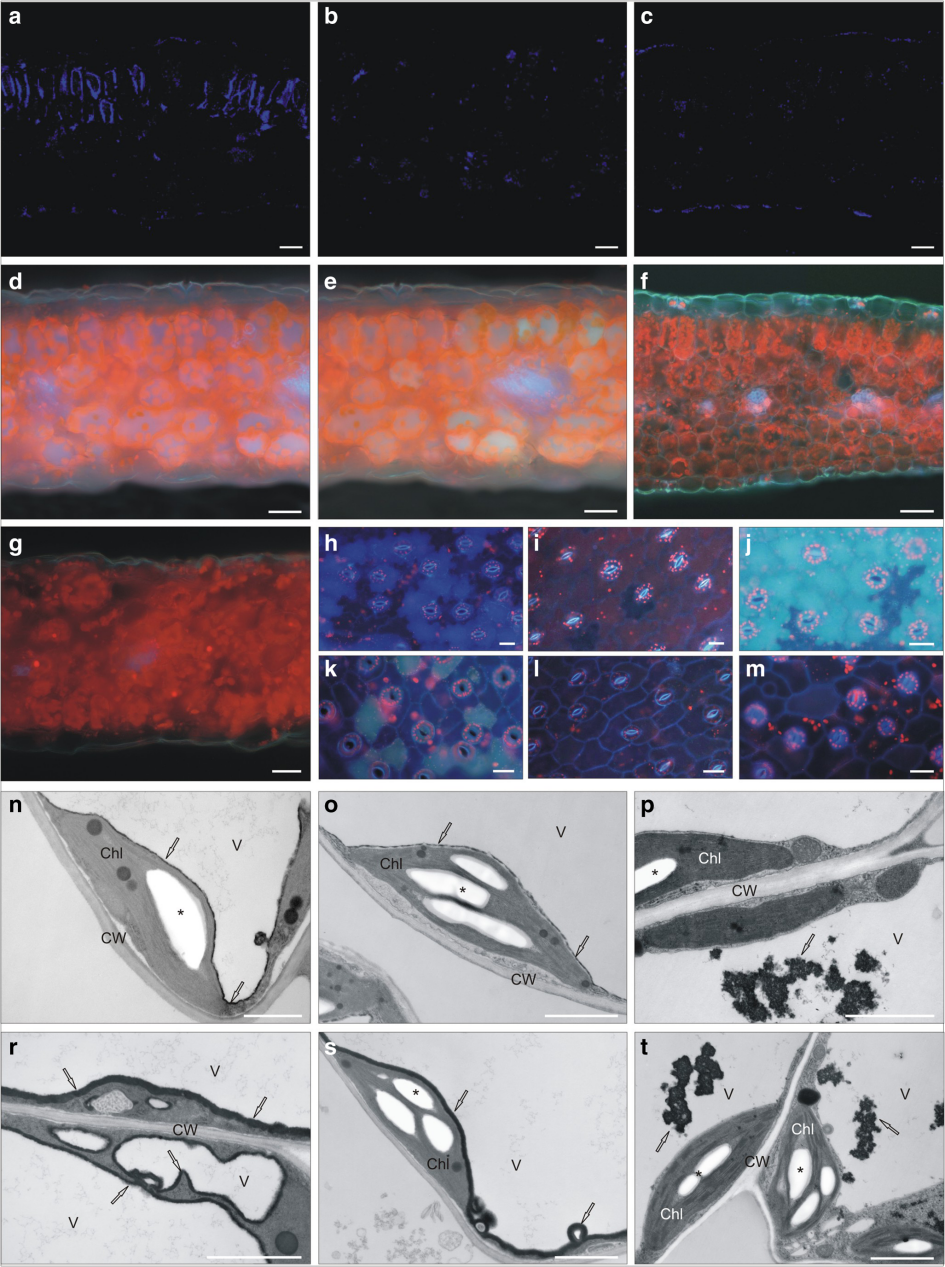
Localization of non-enzymatic antioxidants generated in *Silene vulgaris* leaves under long-term Pb stress. **a**–**c** Reduced glutathione detection in palisade parenchyma of calamine leaves (**a**), and sporadically in serpentine (**b**) and non-metallicolous (**c**) mesophyll cells. Staining with monochlorobimane as fluorescent marker. **d**–**m** Autofluorescence of secondary metabolites. Blue (**d**) and green (**e**) autofluorescence of vacuoles in non-metallicolous leaves. Red autofluorescence of vacuoles, blue-green emission of cell walls, and pink autofluorescence of phloem in calamine leaves (**f**). Red autofluorescence of vacuoles and spherical structures in serpentine leaves (**g**). The blue (**h**), red (**i**), and green (**j**) autofluorescence of vacuolar content of lower epidermal cells in non-metallicolous, calamine, and serpentine leaves, respectively. Weak fluorescent signals in upper epidermal cells of non-metallicolous (**k**), calamine (**l**), and serpentine (**m**) leaves. Bar = 25 μm. **n**–**p**, **r**–**t** Ultrastructural phenolic visualization. Phenol accumulation in untreated non-metallicolous (**n**), serpentine (**o**), and calamine (**p**) mesophyll cells visualized as a dark, electron dense precipitates (arrow). The intensive condensation of phenolics around the tonoplast of non-metallicolous (**r**) and serpentine (**s**) specimens. Dark droplets and precipitates inside the vacuoles of calamine cells (**t**). Bar = 2 μm. Chl chloroplast, CW cell wall, V vacuole, asterisk starch grain, arrow phenol precipitates

At the beginning of experiment, low fluorescent signals indicating reduced glutathione (GSH) localization were observed in leaves from all treatments. The presence of GSH molecules, reflected in the fluorescence intensification, was detected only in the cells of palisade mesophyll for CAL individuals treated with Pb^2+^ by 4 weeks (Fig. 6a), while for the other ecotypes, the level of this compound was quite low, comparable, and independent of the stressor presence (Fig. 6b, c).

#### Tissue localization of secondary metabolite

In control specimens of all tested ecotypes, the blue autofluorescence of secondary metabolites was observed in vacuoles on the whole cross-section of metallicolous leaves or in palisade parenchyma cells of NM one. After the application of Pb^2+^, the blue autofluorescence was found in the vacuoles of both parenchyma types in NM leaves (Fig. 6d), in which green fluorescence also occurred (Fig. 6e). Blue autofluorescence was sporadically detected in CAL vacuoles which emitted mostly red signals (Fig. 6f). Additionally, the blue-green emission was noted in the CAL cell walls. In this ecotype, pink autofluorescence of phloem and aquamarine fluorescence of epidermis surface was also demonstrated (Fig. 6f). Moreover, the red autofluorescence was found in other ecotypes; however, in NM and SER mesophyll, it was detected in spherical structures of different sizes emitting much more intensive signals than the whole vacuoles (Fig. 6g).

Furthermore, UV irradiation revealed the pronounced differences in autofluorescence of secondary metabolites from leaf epidermis. The blue, red, and green autofluorescence of vacuolar content of lower epidermal cells was observed in Pb-treated NM, CAL, and SER leaves, respectively (Fig. 6h–j). On the contrary, upper epidermal cells of all tested specimens under Pb^2+^ stress (Fig. 6k–m) as well as both epidermis of NM and SER control leaves showed weak sporadically observed autofluorescence. In case of CAL untreated leaves, yellowish and bright red signals of vacuoles were detected for upper and lower epidermis, respectively.

#### Ultrastructural phenolic visualization

The presence of phenols inside the vacuoles of control leaves were observed sporadically in all tested ecotypes (Fig. 6n–p). After Pb^2+^ application, phenol accumulation visualized as a dark, electron dense substance was intensified in NM (Fig. 6r) and SER cells (Fig. 6s), while in CAL cells, their level did not differ between treated and untreated leaves (Fig. 6t). What is more, the condensation of phenolics in NM and SER specimens was localized around the tonoplast (Fig. 6r, s), whereas in CAL ecotypes, dark droplets and precipitates inside the vacuoles occurred (Fig. 6t).

#### Secondary metabolite concentration

The shoots of CAL ecotype untreated with Pb^2+^ accumulated the highest content of various secondary metabolite groups, which amounts significantly decreased after Pb treatment (Table 2). The exception was total phenol concentration which constant level reached about 368–373 mg 100 g^−1^ FW and was independent on the stressor presence. On the contrary, the concentration of total secondary metabolites, total phenols, and flavonols in NM and SER shoots cultivated on control medium was similar to each other and increased after Pb application. The anthocyanin content in these ecotypes was not altered in stress conditions, while in CAL shoots, their accumulation was reduced significantly. The concentration of phenylpropanoids differentiated particular ecotypes the most. Regardless of treatment, their level in shoots of metallicolous ecotypes was significantly higher than in NM one. Therefore, the phenylpropanoids as well as total secondary metabolite content depended on both ecotype and Pb treatment (medium) taken separately. Additionally, the concentration of all phenolic groups depended on both ecotype and the medium as well as interaction between these factors.

**Table 2:** The phenolic compound content [mg 100 g^−1^ FW] in shoots of non-metallicolous (NM), calamine (CAL), and serpentine (SER) ecotypes of *Silene vulgaris* after 4 weeks of cultivation in the presence of Pb ions

Ecotype	Treatment	Total secondary metabolites	Total phenols	Phenylpropanoids	Flavonoids	Anthocyanins
NM	Control	750.59 ± 25.36 d*	358.01 ± 25.38 bc	206.27 ± 2.76 e	271.59 ± 27.24 c	103.58 ± 21.19 bc
	33 μM Pb	1128.81 ± 12.15 b	502.65 ± 67.99 a	290.13 ± 17.81 c	361.11 ± 26.86 b	126.69 ± 32.79 bc
CAL	Control	1354.02 ± 54.95 a	373.15 ± 22.40 b	382.58 ± 13.28 a	447.49 ± 36.25 a	200.94 ± 37.96 a
	33 μM Pb	947.58 ± 96.03 c	368.84 ± 43.23 b	301.48 ± 3.07 c	287.89 ± 39.99 c	69.95 ± 47.27 c
SER	Control	819.62 ± 35.92 d	286.62 ± 24.95 c	242.34 ± 18.99 d	276.21 ± 17.98 c	122.45 ± 6.33 bc
	33 μM Pb	1107.65 ± 48.98 b	532.87 ± 55.29 a	331.16 ± 12.37 b	387.10 ± 9.89 b	136.43 ± 15.09 b

*Data present means ± SD. Different letters indicate means that are significantly different at *P* < 0.05 according to two-way ANOVA and post hoc test

### Photosynthetic apparatus of *Silene* ecotypes treated with Pb ions

#### Pigment content

In NM shoots, the concentration of chl *a* and *b* significantly decreased about 13–14% under Pb treatment in comparison to untreated shoots (Table 3). Nevertheless, the chl *a*/*b* ratio did not change under stress condition, but it reached the lowest value from all tested ecotypes. On the contrary, chlorophyll content in CAL and SER shoots regenerated on medium supplemented with Pb ions slightly increased or unchanged relative to their control cultures. Moreover, in both metallicolous ecotypes, chl *a*/*b* ratio was higher than in NM one (Table 3). Considering carotenoid content, the differences between treatments within particular ecotypes were statistically insignificant. Furthermore, the ratio of total chlorophylls to carotenoids decreased in NM and CAL shoots cultivated on medium with Pb^2+^, while in SER one, it remained constant. Taking into account statistically significant influence of treatment on total chlorophylls and chl *a* content, chosen parameters of chl *a* fluorescence were evaluated.

**Table 3:** Photosynthetic pigment content [mg g^−1^ FW] in shoots of non-metallicolous (NM), calamine (CAL), and serpentine (SER) *Silene vulgaris* ecotypes after 4-week cultivation in the presence of Pb ions

Ecotype	Treatment	chl *a*	chl *b*	chl *a*/*b*	car	chl *a* + *b*/car
NM	Control	0.78 ± 0.01 a*	0.22 ± 0.01 a	3.55 ± 0.12 b	0.17 ± 0.02 a	5.29 ± 0.26 b
	33 μM Pb	0.66 ± 0.07 b	0.19 ± 0.02 b	3.47 ± 0.19 b	0.16 ± 0.01 ab	5.31 ± 0.31 b
CAL	Control	0.63 ± 0.07 bc	0.16 ± 0.03 bc	3.93 ± 0.41 a	0.12 ± 0.01 c	6.62 ± 1.29 ab
	33 μM Pb	0.64 ± 0.01 bc	0.16 ± 0.02 bc	4.00 ± 0.49 a	0.10 ± 0.03 c	8.17 ± 1.52 a
SER	Control	0.52 ± 0.01 d	0.13 ± 0.02 c	4.00 ± 0.44 a	0.13 ± 0.01 bc	5.20 ± 0.39 b
	33 μM Pb	0.57 ± 0.04 cd	0.14 ± 0.02 bc	4.07 ± 0.33 a	0.14 ± 0.03 abc	5.21 ± 0.25 b

*Data present means ± SD. Different letters indicate means that are significantly different at *P* < 0.05 according to two-way ANOVA and post hoc test

#### Chlorophyll *a* fluorescence

Despite the measure of forward electron transport, the significant effect of the ecotype and the treatment on all parameters of chl *a* fluorescence and additionally the combination of these factors on activity of the water-splitting complex was indicated. Even though the NM ecotype was characterized by the highest chl *a* content, the disturbances in the photosynthetic reactions seemed to be the greatest, which was indicated by the lowest values of *F*_v_/*F*_m_, PI, and RC/ABS (Table 4). Addition of Pb^2+^ to the medium was associated with alteration of PSII in CAL ecotype, in which maximum quantum efficiency of PSII together with activity of the water-splitting complex and performance index decreased. In SER ecotype, Pb treatment contributed to significant diminish of reaction center density per chlorophyll and performance index in comparison to control culture (Table 4).

**Table 4:** The parameters of chlorophyll *a* fluorescence in leaves of non-metallicolous (NM), calamine (CAL), and serpentine (SER) ecotypes of *Silene vulgaris* after 4 weeks of cultivation in the presence of Pb ions

Ecotype	Treatment	*F*_v_/*F*_m_	Area	RC/ABS	*F*_v_/*F*_o_	(1 − *V*_j_)/*V*_j_	PI
NM	Control	0.814 ± 0.008 b*	23,901 ± 8162.659 a	0.636 ± 0.139 bc	4.401 ± 0.404 c	0.484 ± 0.067 b	1.417 ± 0.628 c
	33 μM Pb	0.818 ± 0.010 b	19,431 ± 2827.276 a	0.593 ± 0.046 c	4.627 ± 0.284 bc	0.488 ± 0.023 b	1.367 ± 0.163 c
CAL	Control	0.842 ± 0.002 a	21,327 ± 3439.985 a	0.851 ± 0.087 a	5.346 ± 0.198 a	0.551 ± 0.016 a	2.516 ± 0.363 a
	33 μM Pb	0.826 ± 0.003 b	19,429 ± 2011.288 a	0.821 ± 0.026 a	4.776 ± 0.384 b	0.558 ± 0.007 a	2.084 ± 0.115 b
SER	Control	0.844 ± 0.01 a	23,129 ± 1788.488 a	0.823 ± 0.071 a	5.416 ± 0.256 a	0.556 ± 0.028 a	2.491 ± 0.235 a
	33 μM Pb	0.839 ± 0.009 a	22,434 ± 3039.068 a	0.701 ± 0.066 b	5.221 ± 0.270 a	0.521 ± 0.026 ab	1.921 ± 0.329 b

*Data present means ± SD. Different letters indicate means that are significantly different at *P* < 0.05 according to two-way ANOVA and post hoc test

#### Chloroplast ultrastructure

**Fig. 7:**
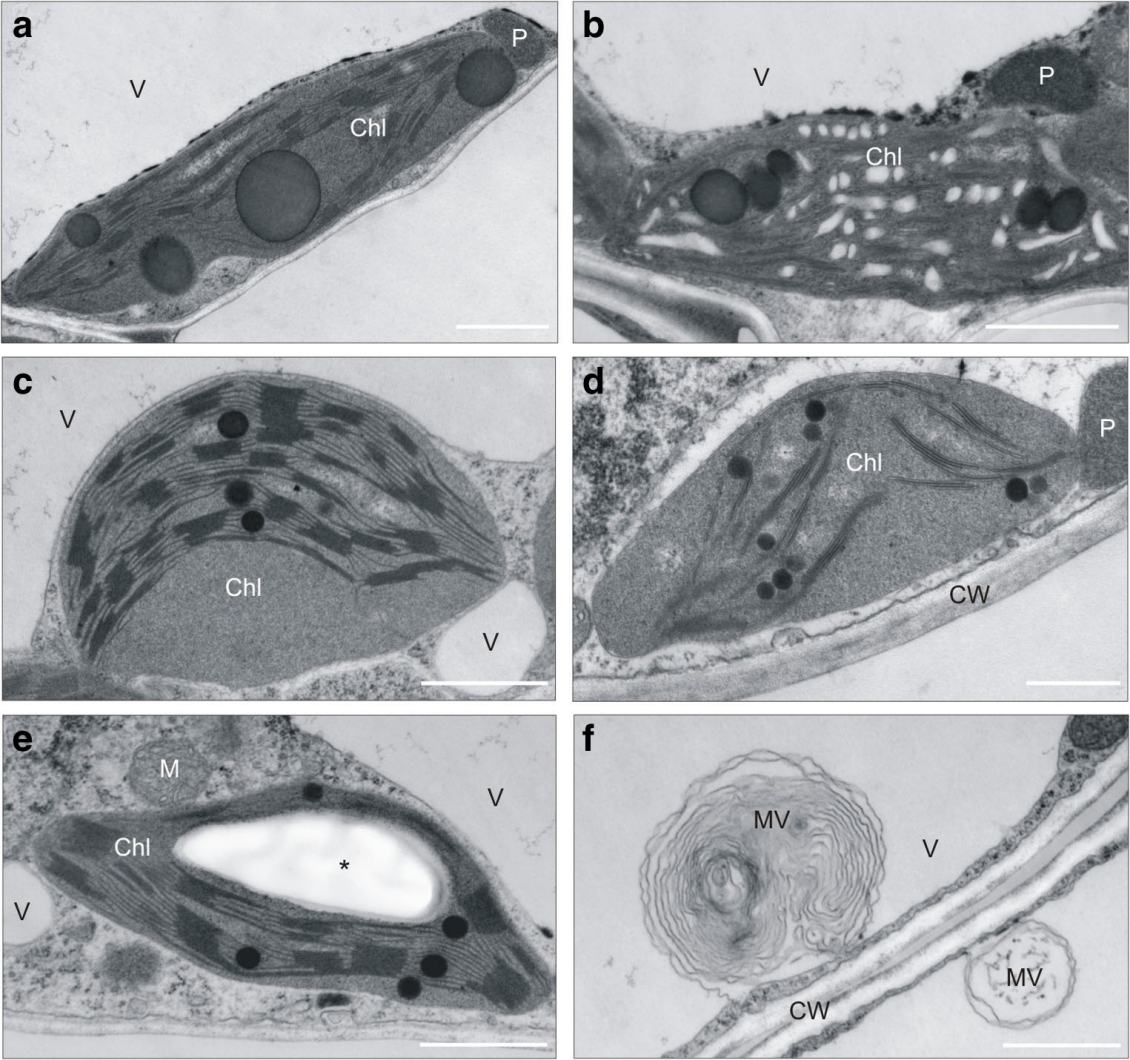
Transmission electron micrograph of leaf mesophyll cells in *Silene vulgaris* specimens cultivated for 28 days on medium enriched with Pb ions. Irregular shape of chloroplast with huge plastoglobuli (**a**) and dilatated thylakoids (**b**) observed in leaves of non-metallicolous specimens. The chloroplasts with swollen stroma (**c**) and looser arrangement (**d**) observed in leaves of serpentine specimens. The regular structure of chloroplasts with starch grain (**e**) and microvesicles and invaginations into vacuoles in calamine specimen cells (**f**). Bar = 1 μm. Chl chloroplast, CW cell wall, M mitochondrion, MV microvesicle, P peroxisome, V vacuole

Transmission electron microscopy (TEM) analysis revealed clear diversification of chloroplast ultrastructure. Generally, in cells of leaves from NM microcuttings, more smaller chloroplasts were noticed in comparison with CAL and SER ones. Regardless of ecotypes, chloroplasts from microcuttings growing on control medium had a regular structure of thylakoids and stroma with numerous starch grains (Fig. 6n–p). Moreover, in chloroplasts of NM and SER leaves, a few small plastoglobules were observed. Medium supplementation with Pb^2+^ reduced the number of starch grains and influenced on chloroplast arrangement (Fig. 7). In NM leaves, the huge plastoglobules appeared in chloroplasts of both parenchyma types (Fig. 7a), and dilatated thylakoids were observed more often than in metallicolous ones (Fig. 7b). In SER leaves, the main chloroplast alternation referred to swollen stroma and a looser membrane arrangement; however, such modification was detected sporadically (Fig. 7c, d). Furthermore, in cells of SER microcuttings treated with Pb^2+^, irregularly shaped mitochondria appeared rarely (Fig. 7d). On the contrary, CAL chloroplast did not show any ultrastructural disorders; however, their size was slightly reduced (Fig. 7e). In this ecotype, the most pronounced changes as compared to respective control culture concerned the occurrence of the numerous vesicles and small vacuoles (Fig. 7f).

## Discussion

Plant communities that spontaneously appear on metal-enriched environments are unique because of their ability to cope with excess amounts of heavy metals (HMs) in the ground. Metallophytes might represent a valuable plant material for phytoremediation and reclamation of chemically degraded areas (Ciarkowska et al. 2017; Muszyńska et al. 2018). In order to develop a new strategy for soil cleanup with biological methods, it is important to understand the basic processes of metal tolerance at cellular and tissue level (Muszyńska et al. 2019). In our study, we used fully controlled, unified in vitro conditions to evaluate the straightforward effect of toxic ions on the status of the same species but originating from various ecological niches. We deliberately applied shoot culture as a model in which by the assumption shoots are deprived roots, and therefore, they could be directly exposed to toxic element. The applied doses of Ni^2+^ and Pb^2+^ corresponded to soluble content of these elements in a place of natural occurrence of serpentine and calamine ecotypes, respectively (Koszelnik-Leszek 2007; Muszyńska et al. 2017). Despite it, the addition of Ni^2+^ to the propagation medium affected growth and development of tested specimens and finally contributed to their death. The negative influence of Ni^2+^ on plants depends inter alia on metal concentration, exposure time, or plant species (Assunçao et al. 2003). In our experiments, TEM analysis revealed the earliest appearing disintegration of cell organelle and membrane disruption in NM shoots cultivated for 24 h on medium enriched with Ni^2+^. In metallicolous ecotypes, the visible symptoms of Ni toxicity as yellowing of leaves were observed later and increased with exposure time. Leaf discoloration might be attributed not only to chloroplast degradation but also to disturbances in photosynthetic pigment synthesis due to Ni-induced deficiency of Mg and Fe involved in this process (Ghori et al. 2019) or from oxidative stress resulting in the membrane lipid peroxidation (Rajpoot et al. 2016). Similar effects were observed for rooted shoots, although it is well-known that root tissue, especially endodermis, limits the supply of HMs such as Cd and Pb to the above-ground parts (Baranowska-Morek and Wierzbicka 2004; Yuan et al. 2015). Nevertheless, Ni^2+^ might be transported to central cylinder either through permeable for them endodermis or through the influx from apical root parts where the Casparian strips have not been yet developed (Seregin and Kozhenikova 2008). It could be confirmed in our experiment in which symptoms of Ni toxicity were observed even if shoots were previously rooted.

Interestingly, in established experimental scheme, also serpentine ecotype of *S. vulgaris* that was considered as tolerant to Ni ions died in their presence. The latest studies underlay the importance of rhizosphere microorganisms in increasing of plant survival under heavy metal stress (Schützendübel and Polle 2002; Becerra-Castro et al. 2012; Yang et al. 2019). Such bacterial communities have been recently described also for *S. vulgaris* from areas contaminated with chromium (García-Gonzalo et al. 2017) or lead, zinc, and cadmium (Pacwa-Płociniczak et al. 2018). Thus, it is highly probable that tested ecotypes growing on artificial medium in aseptic conditions were deprived positive microbes that may influence on metal mobility in natural environment. It makes an opportunity for further research on this plant species-microbial interactions and possible culture biotization to improve micropropagated plant resistance to abiotic stress.

Clonal propagation of CAL and SER ecotypes on Pb-containing medium was just as efficient as on medium without Pb ions. It was reflected in growth tolerance index which ranged nearly 150% for CAL and 100% for SER specimens. The enhanced tolerance of these ecotypes to Pb treatment could be related to increase in dry weight of shoots, as reported in the cultures of *Populus alba* (di Lonardo et al. 2011) or *Daphne jasminea* (Wiszniewska et al. 2017). On the contrary, multiplication of NM culture was significantly inhibited in the presence of Pb^2+^. An appreciable decline in morphogenesis under Pb stress has been also observed in *Holarrhena antidysenterica* (Agrawal and Sharma 2006) or *Alyssum montanum* (Muszyńska et al. 2018) cultured in vitro. Thus, the obtained results indicate definitely lower resistance of *S. vulgaris* from non-contaminated area to HMs than ecotypes from post-industrial terrains. The NM culture growth response corresponded to the increasing accumulation of HMs in their tissue. In this ecotype, metals were visualized in the vacuoles on the whole leaf blade cross-section, while in metallicolous ones, ions were localized more precisely in the cytoplasm (CAL) or in the vacuoles of spongy (CAL) and palisade parenchyma (SER). The maintenance of ion homeostasis in cells is crucial for sustainable plant growth and development (Ovečka and Takáč 2014; Yuan et al. 2015). Therefore, when organisms are exposed to high concentrations of HMs, the activation of intracellular mechanisms, which allow them to avoid deleterious effects of metal toxicity, is particularly important. Our research demonstrated that in metallicolous *Silene* ecotypes, detoxifying strategies consist mainly in HMs removal from metabolically active cytoplasm by moving them into inactive compartments. Such compartmentation can refer to cell walls, vacuoles of leaf mesophyll, and epidermis, the trichomes, or the cuticle (Baranowska-Morek and Wierzbicka 2004; Rascio and Navari-Izzo 2011; Tian et al. 2011; Muszyńska et al. 2018). Nevertheless, HMs were not detected in *Silene* organs at ultrastructural level. Probably the time of Pb treatment was too short to incorporate the toxic ions into cell walls, and therefore, they remained dissolved only in water cell phase, which was removed during the fixation for TEM observations. Such results are not consistent with the study of Baranowska-Morek and Wierzbicka (2004) that revealed black precipitates in cells of *Dianthus carthusianorum* roots after similar incubation time and Pb dose. However, in this case, ultrastructural Pb detection was not combined with energy-dispersive X-ray spectrometry as in our examination. Unlike SER ecotype, in CAL specimens, metal accumulation increased towards the edge of leaf blade. Considering SEM-EDX analyses of metal content, which showed the highest concentration of Pb^2+^ in CAL trichomes located on leaf edges, the results suggest the ability of this ecotype to remove and accumulate the toxic ions in these epidermal structures. Moreover, in CAL leaves, fluorescent signals indicating metal presence were also detected in the cytoplasm of palisade cells. It may implicate another mechanism based on metal binding with organic ligands in the cytoplasm (Maestri et al. 2010; Kumar and Prasad 2018). Since microscopic visualization of GSH revealed its localization in the same palisade cells, it is highly probable that Pb-GSH complexes were formed in *Silene* leaves to protect plants from deleterious effects of HMs, as in, e.g., *Vetiveria zizanioides* (Andra et al. 2009), *Sedum alfredii* (Tian et al. 2011), or *Iris lactea* (Yuan et al. 2015). It was found that such Pb complexes can be either transferred into the vacuole or served as a donor of metallic ions to stronger metal chelators—metallothioneins and phytochelatins (Yadav 2010; Kumar and Prasad 2018). Taking into account studies on Cu-tolerant plants of *S. vulgaris* (van Hoof et al. 2001) or *S. paradoxa* (Mengoni et al. 2003) showing enhanced accumulation of metallothioneins in their leaves, in tested CAL ecotype, Pb ions might be complexed directly or via GSH with these metal-binding proteins. Therefore, our finding suggests the genotype-specific mechanisms for coping with toxic elements by *S. vulgaris* specimens from various habitats.

A common consequence of HM stress is overproduction of ROS. Similarly, the results of our study indicated intensified ROS accumulation in HM-treated leaves; however, their level depended on ecotype and duration of culture. Under short-time stress (24 h), the highest ROS level was detected in CAL and SER leaves, while in NM leaves, ROS level was significantly lower than in other ecotypes. Under long-term cultivation in the presence of Pb^2+^ (4 weeks), the overall ROS accumulation exhibited the opposite tendency and changed as follows: NM > SER ≥ CAL. Overproduction of ROS has been observed earlier in many other species under HM stress (Nahar et al. 2016; Rajpoot et al. 2016; Muszyńska et al. 2018). Thus, for a long time, ROS have been considered as dangerous molecules, whose levels need to be kept as low as possible (Schützendübel and Polle 2002; Manquián-Cerda et al. 2018; Ghori et al. 2019). Recently, it has been also realized that ROS play an integral role as signaling molecules in the regulation of numerous biological processes and plant responses to abiotic and/or biotic stresses (Miller et al. 2008; Baxter et al. 2014; Rajpoot et al. 2016; Waszczak et al. 2018). Despite it, there are no literature data on ROS metabolism in metallophytes. Based on present research, the oxidative burst observed just 24 h after HMs application might suggest the ROS involvement in temporal-spatial coordination of signals in metallicolous *Silene* ecotypes that result in activation of mechanisms preventing further damage caused by long-term exposition to HMs. Nevertheless, the molecular and biochemical basis of such signal transduction pathways require further examinations. On the contrary, in NM specimens, no ROS was initially detected which finally led to their enhanced accumulation and morphological and ultrastructural disturbances in this ecotype. Considering our findings on H_2_O_2_ visualization, which showed its abundant presence after 24 h in the cells of CAL and SER shoots from Pb- and Ni-enriched medium, respectively, and comparable H_2_O_2_ level between their treated and untreated shoots after 4 weeks, we can assume the potentially important role of H_2_O_2_ in response of metallicolous ecotype to these metals to which they have been adapted.

An effective marker of cellular oxidative damage that resulted from enhanced ROS accumulation is lipid peroxidation (Rajpoot et al. 2016; Emamverdian et al. 2018; Waszczak et al. 2018). In our experiment, increased production of TBARS in Pb-treated shoots, showing increased lipid peroxidation, was noted in all tested *Silene* cultures after 4 weeks of cultivation. However, the rise in TBARS level seems to depend on ecotype. Taking into account enhanced ROS generation in NM specimens, the observed lipid peroxidation suggests the Pb-induced oxidative stress in its shoots. Therefore, the appearance of numerous large plastoglobules in chloroplasts might indicate ROS and/or Pb influence on membrane structures. In turn, looser arrangement of thylakoids could be contributed to the reduction of chl *a* content since this molecule is crucial for grana formation and stabilization. Despite it, the blockage of photosynthetic apparatus efficiency and PSII functioning was negligible, although calculated parameters of chl *a* fluorescence slightly diminished under Pb^2+^ influence. It is highly probable that the reduction of photosynthetic pigment concentration might be attributed to the disturbances in the chlorophyll synthesis due to the inhibition/degradation of enzyme activities that are involved in CO_2_ fixation (Sandalio et al. 2001; Kumar and Prasad 2018). On the other hand, the plastoglobules are a pool of lipids such as tocopherols and carotenoids that play a role as both antioxidants in ROS quenching (Otsubo et al. 2018) and chloroplast stabilization (Rottet et al. 2015). We can not exclude that their presence in NM leaves might suggest the activation of ROS defense mechanisms and/or remodeling of membrane lipids leading to the acclimatization of photosynthetic apparatus to stress conditions. In metallicolous ecotypes, such adaptation was expressed in higher chl *a/b* ratio than in NM one.

The level of lipid peroxidation similar to NM Pb-treated specimens was also ascertained in CAL culture. However, CAL leaves were simultaneously characterized by a cessation of photosynthesis, reduction in chloroplast size, invagination of the tonoplast, and occurrence of small vesicles. All these events take place during premature senescence induced by HM toxicity as observed among others in *Pisum sativum* (Sandalio et al. 2001), *Camellia sinensis* (Mukhopadhyay et al. 2013), and *Alyssum montanum* (Muszyńska et al. 2018). Nevertheless, studies conducted by Wierzbicka and Panufnik (1998) and Muszyńska et al. (2019) on metal-tolerant *S. vulgaris* specimens showed the r-type life strategy in this species. It means that plants exhibit fast growth and development, a short life cycle, and large reproductive efforts. Such traits give a greater chance for the survival in harsh environment. Therefore, observed in our experiment, senescence symptoms result from intensive multiplication rate rather than metal toxicity and may confirm the adaptation of CAL ecotype to Pb ions.

ROS levels in a plant cell are under tight control of defense systems that include GSH and phenols (Kumar and Prasad 2018). In our study, GSH was localized only in CAL Pb-treated leaves, which may implicate its dual role in this ecotype both as HM binding and ROS scavenging. Therefore, we focused mostly on secondary metabolites which were visualized by UV light excitation. This microscopic method does not require any previous treatment and provides an effective experimental tool for distinguishing many endogenous fluorophores (Talamond et al. 2015). Autofluorescence of various compounds may serve as natural indicator of cellular state since the emission often shows changes in metabolism and responses to various stressors (Roshchina 2012; Yuan et al. 2015; Vidot et al. 2019). In our study, the blue and green autofluorescence dominated in the vacuoles of Pb-treated NM leaves and in epidermis of SER ones. The blue autofluorescence of secondary metabolites belongs to phenolics, alkaloids, coumarins, and/or pterines, while green one indicates the presence of flavonoids and/or terpenoids (Monici 2005; Roshchina 2012). The red autofluorescence emission is generally attributed to the chloroplasts which contain strongly fluorescent chlorophylls (Buschmann et al. 2000; Vidot et al. 2019). However, it may be also an indicator of anthocyanins, anthocyanidins, and azulene presence in the tissue (Borucki and Sujkowska 2008). Anthocyanins are predominantly stored in the central vacuoles of most plants, and such accumulation type was observed mostly in CAL *Silene* ecotype. Moreover, they can be found as an anthocyanic vacuolar inclusions (AVIs) or anthocyanoplasts (Pecket and Small 1980; Pourcel et al. 2010). The appearance of additional structures filled with anthocyanins in SER and NM specimens fits well to abovementioned anthocyanin-containing bodies. Thus, our results suggest diversified pathways of anthocyanin accumulation, although their concentration in particular ecotypes did not differ significantly between treatments. Anthocyanins together with flavonols belong to flavonoids, which role in ROS spreading relies not only on direct ROS scavenging but also on cell membrane stabilization by interaction with their compounds that restrict ROS diffusion (Arora et al. 2000; Karuppanapandian et al. 2011; Biesiada and Tomczak 2012; Muszyńska et al. 2019). This defense mechanism seems to be unspecific for particular *Silene* ecotypes, since concentration of these phenolic groups increased comparatively in NM and SER specimens treated with Pb^2+^. In turn, the phenylpropanoid content in metallicolous ecotypes was significantly higher than in NM ones. It implicates the activation of lignin’s synthesis pathways, especially that the blue-green emission of cell walls in CAL leaves was also observed. Such signal localization suggests the presence of hydroxycinnamic acid which is an intermediate in the lignin synthesis (Talamond et al. 2015). By lignin deposition within cell walls, the more effective mechanical barrier against HM penetration into the protoplast is formed, which has been confirmed in *Matricaria chamomilla* (Kováčik and Klejdus 2008) or *Glycine max* (Pawlak-Sprada et al. 2011) exposed to HMs. Thus, it is highly probable that prevention of enhanced ROS generation and accumulation in metallicolous ecotypes bases also on the extracellular barrier development that restrict penetration of factors responsible for oxidative stress. Moreover, TEM analysis revealed diversified pattern of phenolic accumulation inside the vacuoles in tested ecotypes that may confirm the presence of different pathways of phenol synthesis in non-metallicolous and metallicolous specimens.

## Conclusions

In present research, the responses of *Silene vulgaris* to Ni and Pb ions were described in details and linked with potential strategy to cope with metal toxicity. The mode of HM actions was compared between representatives of the same species but originating from three contrasting populations. Although Ni application contributed to shoot cultures’ death, the study revealed that the mechanisms of Pb detoxification differ between non-metallicolous and metallicolous ecotypes. In both CAL and SER specimens, the accumulation of phenylpropanoids involved in lignin’s biosynthesis was observed, and therefore, the formation of thicker and more effective mechanical barrier against HM penetration seems to be unspecific reaction of metallicolous ecotypes. Interestingly, Pb presence in the protoplasts lead to the activation of ecotype-specific intracellular mechanisms that resulted in ion compartmentation in palisade mesophyll of SER leaves or in trichomes of CAL one, in which complexation with glutathione can also help reduce metal toxicity. Such mechanisms of avoiding the negative Pb effects were not found in NM specimens. Moreover, both metallicolous ecotypes exhibited increased ROS production in leaves, even before the harmful ions got into these parts of plants. It may implicate the potential role of ROS in activation of defense mechanism leading to the synthesis of low molecular non-enzymatic antioxidants that were diversified in time and in respective ecotypes. Therefore, the response of SER and CAL ecotypes at morphological, physiological, and ultrastructural level was almost unchanged under Pb treatment, albeit CAL specimens showed accelerated growth and thus senescence symptoms. On the contrary, the reaction of NM ecotype to excess amounts of Pb ions resulted in typically observed excess ROS generation, growth disturbances, and chlorophyll degradations. To our knowledge, the present findings for the first time indicate on integral function of ROS as a signaling molecule in metal-tolerant species and provide a valuable contribution in already extensive knowledge on vascular plant adaptations to increased concentrations of HMs. On the other hand, it creates new possibilities for further research on ROS metabolism and signaling pathways in metallophytes.

## Abbreviations

CAL: Calamine ecotype
HMs: Heavy metals
NM: Non-metallicolous ecotype
SER: Serpentine ecotype
ROS: Reactive oxygen species
